# Excess salt exacerbates blood-brain barrier disruption via a p38/MAPK/SGK1-dependent pathway in permanent cerebral ischemia

**DOI:** 10.1038/srep16548

**Published:** 2015-11-09

**Authors:** Tongshuai Zhang, Shaohong Fang, Cong Wan, Qingfei Kong, Guangyou Wang, Shuangshuang Wang, Haoqiang Zhang, Haifeng Zou, Bo Sun, Wei Sun, Yao Zhang, Lili Mu, Jinghua Wang, Jing Wang, Haiyu Zhang, Dandan Wang, Hulun Li

**Affiliations:** 1 Department of Neurobiology, Harbin Medical University, Harbin, China 150081; 2 The Key Laboratory of Myocardial Ischemia, The Second Affiliated Hospital of Harbin Medical University, Harbin, China 150081; 3 Department of Neurology, The First Affiliated Hospital of Harbin Medical University, Harbin, China 150081; 4 Department of Epidemiology and Biostatistics, Harbin Medical University, Harbin, China 150081

## Abstract

High salt diet (HSD) is one of the most important risk factors that contribute to many vascular diseases including ischemic stroke. One proposed mechanism underlying the disruption of blood-brain barrier (BBB) mediated by HSD is indirectly through enhancing blood pressure. The direct role of HSD on BBB integrity is unclear. Our purpose is to determine whether and how HSD might be involved in BBB breakdown during ischemia. To test that, we induced model of cerebral ischemia by permanent middle cerebral artery ligation (pMCAL) in either normal diet or HSD fed mice. We observed that HSD significantly enhanced ischemic brain damage which was associated with enhanced BBB disruption, increased leukocytes infiltration and loss of tight junction (TJ) proteins expression without apparently altering blood pressure. Our *in vitro* experiment also revealed that sodium chloride (NaCl) treatment down-regulated TJ protein expression by endothelial cells and substantially increased BBB permeability during starvation. Inhibition of p38/MAPK/SGK1 pathway eliminated the effect of NaCl on BBB permeability *in vitro*. In addition, we noticed a positive correlation between urinary sodium levels and ischemic lesion size in stroke patients. Together, our study demonstrates a hypertension-independent role of HSD during ischemia and provides rationale for post cerebral ischemic attack management.

Stroke is a major cause of death and long-term disability worldwide. Acute ischemic stroke, the most common form of cerebrovascular disease, imposes a great economic and social burden due to the physical and mental disability resulting from stroke-related brain damage. Increasing evidences have suggested that sustained excess salt intake is associated with higher risk of stroke, likely by promoting hypertension and fibrosis in the arteries^1^,^2^,^3^,^4^,^5^.

Blood brain barrier (BBB) is the major site of blood–central nervous system (CNS) exchange at the level of brain microvessel endothelium. Brain microvascular endothelial cells (BMECs), the primary structure responsible for BBB permeability^6^,^7^, are connected by tight junctions (TJ) and play an important role in controlling ion homeostasis within the brain^8^,^9^. It has been reported that alterations of TJ-associated proteins contribute to the loss of BBB function in many central nervous system diseases and disorders^10^. Loss of integrity of BBB is one of the key events in ischemic stroke. However, it remains unconfirmed whether a high salt diet directly promotes loss of BBB integrity in stroke development.

Previous studies have demonstrated that serum/glucocorticoid-regulated kinase 1 (SGK1) is a highly conserved and important regulator of the epithelial amiloride-sensitive sodium channel. SGK1 is involved in disorders, including hypertension, ischemia, neurodegeneration and fibrosing diseases etc. Studies have revealed that BMECs are the major sources of SGK1 expression within the brain^11^,^12^. In addition, SGK1 has been identified as a genetic risk factor that is associated with the development of ischemic stroke^13^. Moreover, the up-regulation of SGK1 is mediated by p38 kinase^14^. A recent study has demonstrated that high-salt conditions increased SGK1 expression in naïve CD4^+^ T cells, promoting the development of SGK1-related diseases^3^. It is still not clear whether SGK1 affects the function and structure of the neurovascular endothelium under high-salt conditions.

In this study, we first showed that HSD could enhance ischemic brain damage without changing blood pressure *in vivo*, which was associated with increased BBB permeability as indicated by down-regulation of TJ associated molecules and enhanced immune cells infiltration. Furthermore, our *in vitro* experiments demonstrated that HSD was directly involved in regulating the permeability of BBB through p38/MAPK/SGK1 signaling pathway.

## Results

### Excess dietary salt intake induced severe ischemic brain damage

**Figure 1:**
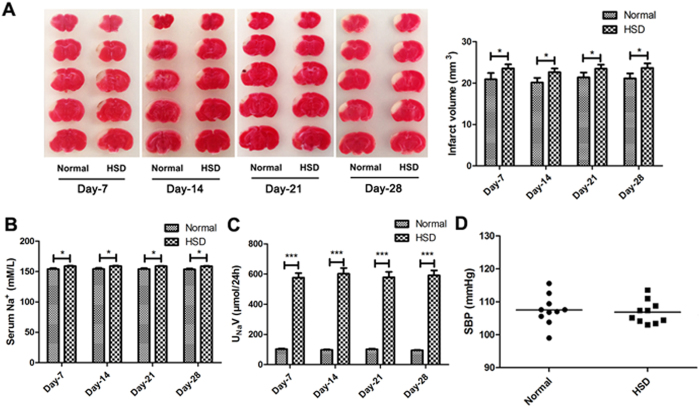
Excess dietary salt intake induced more severe ischemic brain damage. (**A**) Representative TTC-stained brain sections of selected diets-treated mice in different groups were processed 2 days after pMCAL, while the infarct lesion remains unstained and normal brain tissue is stained red. (*P < 0.05, n = 5/group, compared with the corresponding values for the ipsilateral hemispheres). (**B**) Serum sodium concentration and (**C**) 24-hour urinary sodium excretion (U_Na_V) of mice in normal and high-salt diet groups at different time points prior to pMCAL induction are shown. (*P < 0.05, ***P < 0.001, n = 10/group). (**D**) Systolic blood pressure (SBP) of mice fed with normal or high-salt diet for 28 days was measured before pMCAL induction (n = 10/group). Data are pooled from two dependent experiments. Values are mean ± SD.

To test the role of HSD during ischemia, mice were fed with normal diet or HSD for 7 days, 14 days, 21 days or 28 days before permanent middle cerebral artery ligation (pMCAL) was performed. Brain tissues were collected 2 days after the surgery. 2,3,5-triphenyltetrazolium chloride (TTC) staining was used to measure brain damage (Fig. 1A). We found that the brain ischemic area of HSD pretreated mice was significantly increased as compared to the mice fed with normal diet (Day-7: *P* = 0.0277, Day-14: *P* = 0.0141, Day-21: *P* = 0.0343 and Day-28: *P* = 0.0214). 24-hour urinary sodium (U_Na_V) (Fig. 1C) data showed that HSD treated mice had much higher urinary sodium concentration than those with normal diet (*P* < 0.001). While the serum sodium level (Fig. 1B) was only marginally increased (Day-7: *P* = 0.0145, Day-14: *P* = 0.0164, Day-21: *P* = 0.0162 and Day-28: *P* = 0.0154). Moreover, systolic blood pressure (SBP) was measured using a tail-cuff system after 28 days of selected dietary sodium feeding prior to pMCAL. As shown in Fig. 1 D, there was no significant difference in the SBP between the normal and HSD groups (*P* > 0.05).

### HSD down-regulated the expression of tight junction proteins ZO-1 and Occludin and exacerbated BBB breakdown in the pMCAL model

**Figure 2:**
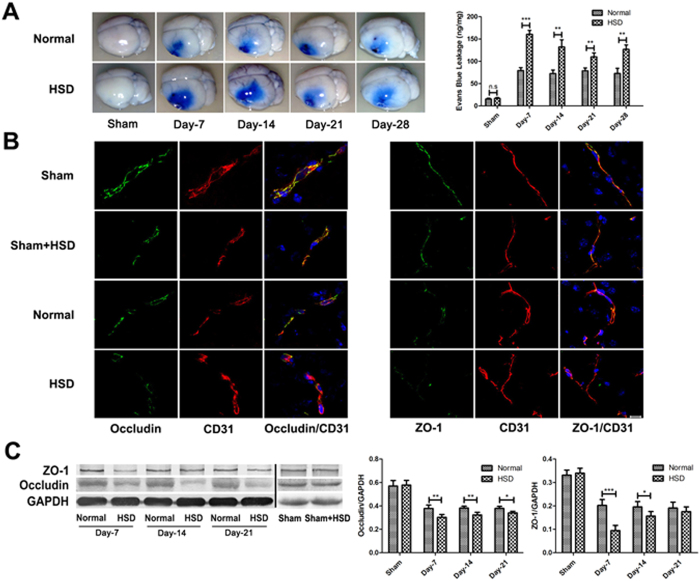
High-salt loading increased BBB breakdown. (**A**) Evans blue leakage was detected 2 days after pMCAL in mice fed with selected diets for 7 days, 14 days, 21 days and 28 days. The brain of the HSD mouse have a larger blue area (left), and higher Evans blue content (bar graph on right) as compared with that of the normal diet mouse (n = 5 per group; **P < 0.01, ***P < 0.001) (**B**) Occludin and ZO-1 were co-stained with CD31, a capillary endothelia marker, in the penumbra region of the ischemic brain of different groups (n = 5 per group). Scale bar, 10 μm. (**C**) High-salt conditions (HSD for 7 days, 14 days, 21 days and 28 days) down-regulates the TJ proteins ZO-1 and Occludin expression 2 days after pMCAL. (n = 5 per group; *P < 0.05, **P < 0.01, ***P < 0.001). Values are mean ± SD.

To test whether HSD might impact the permeability of BBB, we used Evans blue dye (EBD) to demonstrate extent of albumin leakage^15^. As shown in Fig. 2A, EBD leakage was significantly increased in the HSD group as compared to the normal diet group after pMCAL (Day-7: *P* < 0.001, Day-14: *P* = 0.0077, Day-21: *P* = 0.0092, and Day-28: *P* = 0.0083). It has been reported that alterations of TJ-associated proteins such as ZO-1 and Occludin contribute to the loss of BBB function in many central nervous system diseases and disorders^10^. Next, ZO-1 and Occludin expressions in the brain tissue from animals of HSD or normal diet groups were detected using immunofluorescence staining and western blotting. Both ZO-1 and Occludin expressions were down-regulated in CD31-positive capillaries in the ischemic penumbra region (Fig. 2B). HSD further decreased the expression of ZO-1 (Day-7: *P* < 0.001 and Day-14: *P* = 0.0408) and Occludin (Day-7: *P* = 0.0067, Day-14: *P* = 0.0054 and Day-21: *P* = 0.0145) as compared with normal diet group (Fig. 2C), which was also confirmed by western blotting. No significant changes of EBD leakage and TJ molecule expression were observed in the sham operation group fed with either HSD or normal diet. From these experiments, we concluded that HSD increased ischemic brain damage possibly through increasing BBB permeability.

### The infiltration of peripheral immune cells was increased after HSD in the pMCAL model

**Figure 3:**
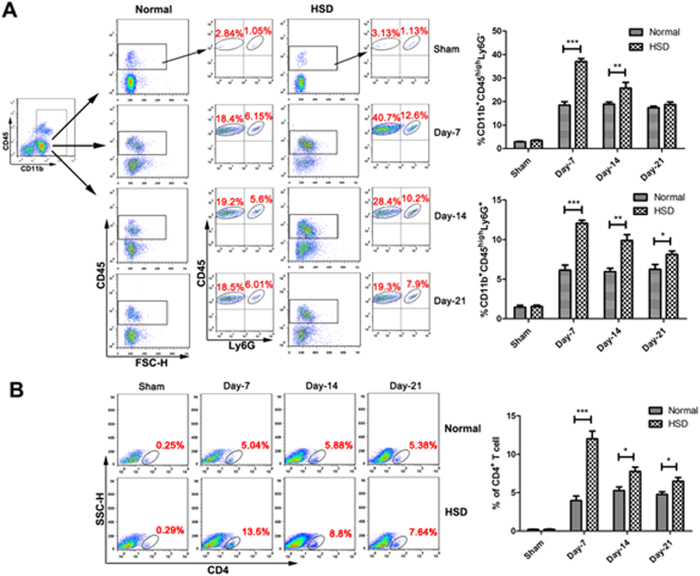
High-salt diet exacerbated infiltration of macrophages, neutrophils and CD4^+^ T lymphocytes in pMCAL. (**A**) Infiltration of macrophages (CD11b^+^CD45^high^Ly6G^−^) and neutrophils (CD11b^+^CD45^high^Ly6G^+^) determined by flow cytometry in the ischemic brain hemispheres of the normal diet and high-salt diet mice. (n = 6 per group; *P < 0.05, **P < 0.01, ***P < 0.001). (**B**) The percentages of infiltrated CD4^+^ T cells lymphocytes in the ischemic brain tissue are shown (Representative data on the left, quantification bar graph on the right). (n = 6 per group; *P< 0.05, ***P < 0.001). Values are mean ± SD.

To further demonstrate that a HSD was able to enhance the BBB permeability after ischemia, we detected the presence of infiltrating inflammatory cells in the brain 2 days after pMCAL induction^16^,^17^. Brain leukocytes were isolated by Percoll gradient centrifugation, and flow cytometry was conducted to evaluate the ratio of infiltrating cells^18^,^19^. As shown in Fig. 3A, the results revealed higher percentages of CD11b^+^CD45^high^Ly6G^−^ cells (peripheral macrophages, Day-7: *P* < 0.001 and Day-14: *P* = 0.0086), CD11b^+^CD45^high^Ly6G^+^ cells (peripheral neutrophils, Day-7: *P* < 0.001, Day-14: *P* = 0.0032 and Day-21: *P* = 0.0426) in mice with the 7 days, 14 days, and 21 days of HSD than those in mice with normal diet. The analysis of the transendothelial migration of CD4^+^ T cells revealed a 3.04-fold increase in the infiltration ratio in the 7 days HSD group compared with that of control mice (Day-7: *P* < 0.001). However, the 14 days and 21 days HSD groups showed a relatively decreased ratio of infiltrating CD4^+^ T cells in the ischemic brains (Day-14: *P* = 0.0186 and Day-21: *P* = 0.0143), which remained 1.51-fold higher in comparison with the control groups (Fig. 3B).

### Sodium chloride down-regulated TJ-associated proteins expression *in vitro*

**Figure 4:**
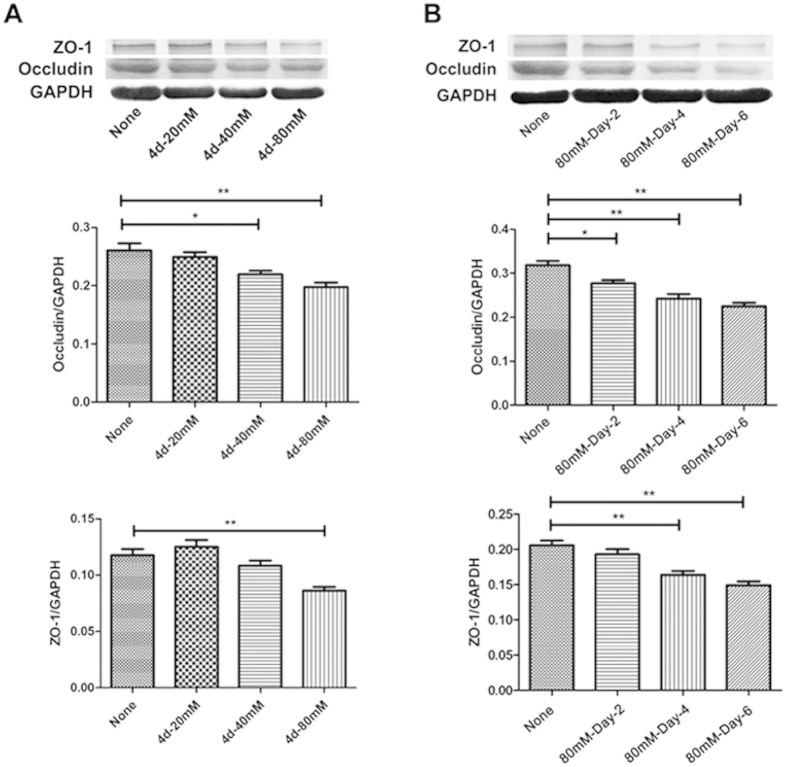
Additional salt stimulation down-regulated the expression of ZO-1 and Occludin in bEnd.3 cultures. (**A**) The expression of ZO-1 and Occludin in bEnd.3 cells after NaCl treatment at various concentrations and (**B**) indicated time points is detected by western blotting (*P < 0.05, **P < 0.01, ***P < 0.001). The data represent are pooled results from four independent experiments. (*P < 0.05, **P < 0.01, ***P < 0.001). Values are mean ± SD.

To investigate the direct effects of HSD on brain microvascular endothelial cells, bEnd.3 cells were treated with different concentrations of NaCl for 2, 4, and 6 days. Expression of TJ-associated proteins ZO-1 and Occludin in the bEnd.3 cell were measured by western blotting. Since cell death was induced by 160 mM treatment, 80 mM of NaCl was selected as an optimal dose (Figure S1), and this concentration further remarkably decreased both ZO-1 (None vs 4 d-80 mM: *P* = 0.0025) and Occludin (None vs 4 d-40 mM: *P* = 0.0245 and None vs 4 d-80 mM: *P* = 0.0047) expression (Fig. 4A). To study the dynamic change of ZO-1 and Occludin expression after NaCl treatment, we measured ZO-1 and Occludin expression in bEnd.3 cell lysates at different time points after NaCl treatment. NaCl started to decrease the expression of both molecules at day 4 and further reduced ZO-1 (None vs 80 mM-Day-4: *P* = 0.0029 and None vs 80 mM-Day-6: *P* = 0.0019) and Occludin (None vs 80 mM-Day-2: *P* = 0.0116, None vs 80 mM-Day-4: *P* = 0.0025 and None vs 80 mM-Day-6: *P* = 0.0021) expression along with time (Fig. 4B). Based on these data, we incubated bEnd.3 cells with 80 mM NaCl for 4 days for the following tests. Additionally, we determined which factor, cations, anions, or osmolarity contributed to the decrease in ZO-1 and Occludin protein production. The addition of 40 mM sodium gluconate caused a similar degree of TJ associated protein expression level as the same NaCl concentration; however, mannitol and MgCl_2_ had no effect (Figure S2), suggesting the sodium cation was critical for ZO-1 and Occludin protein expression and that this sodium ion induced decrease in tight junction related proteins was likely independent of osmotic influences.

### High-salt accelerated oxygen glucose deprivation (OGD)-induced endothelial injuries *in vitro*

**Figure 5:**
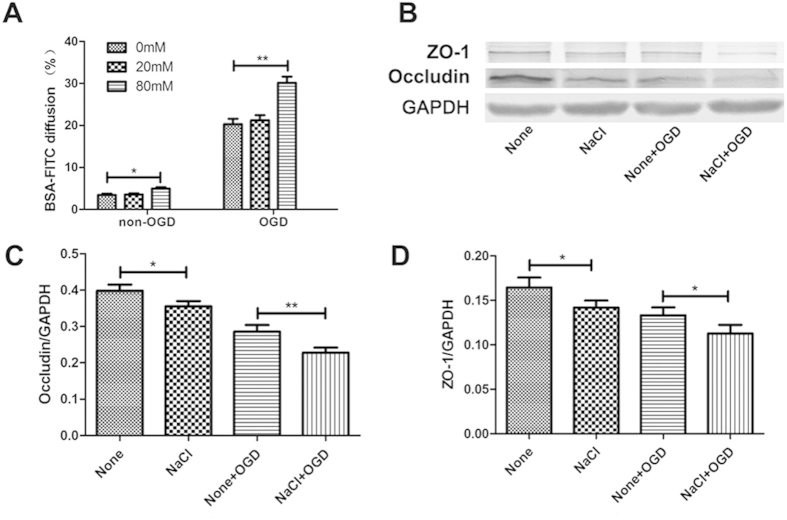
Excess salt intake accelerated OGD-induced BBB disruption associated with ZO-1 and Occludin down-regulation *in vitro.* (**A**) Confluent bEnd.3 cells grown on 24-well cell culture inserts with indicated concentrations of NaCl for 4 days, and then BSA-FITC addition together with 6 h OGD/non-OGD treatments were processed. The bar graph shows the statistical analysis of the BSA-FITC diffusion rate. The data represent are pooled results from three independent experiments. (*p < 0.05, **p < 0.01, ***p < 0.001). (**B**) Representative western blotting of ZO-1 and Occludin expression under high-salt and OGD conditions. The statistical analysis is shown in bar graphs (**C**,**D**). The data represent are pooled results from four independent experiments. (*P < 0.05, **P < 0.01). Values are mean ± SD.

In order to test whether high-salt can directly impact endothelial cell barrier, we cultured bEnd.3 cells with NaCl under normal or starvation condition *in vitro*. bEnd.3 cells were cultured on top of cell culture inserts for 6 h-OGD/non-OGD treatments after 4-days’ treatment of NaCl. The barrier integrity of the bEnd.3 monolayer was assessed by the BSA-FITC transfer rate^20^. As shown in Fig. 5A, 80 mM NaCl alone significantly accelerated the transmission of BSA-FITC through the endothelial monolayer (0 mM vs 80 mM: *P* = 0.0276). Cells exposed to 0 mM NaCl plus non-OGD were set as negative controls. The BSA-FITC diffusion rate was further promoted by the concentration of 80 mM NaCl under OGD incubation compared with 0 mM NaCl after 6 h OGD treatment (0 mM vs 80 mM: *P* = 0.0071). As expected, OGD treated bEnd.3 cells in 80 mM sodium chloride supplemented cultures showed a significant reduction of the expression of ZO-1 (None vs NaCl: *P* = 0.0159 and None + OGD vs NaCl + OGD: *P* = 0.0204) and Occludin (None vs NaCl: *P* = 0.0132 and None + OGD vs NaCl + OGD: *P* = 0.0082) as compared with OGD treated cell cultures without additional sodium supplement (Figs. 5C,D).

### High-salt reduced TJ proteins expression via the p38/MAPK-SGK1 signaling pathway

**Figure 6:**
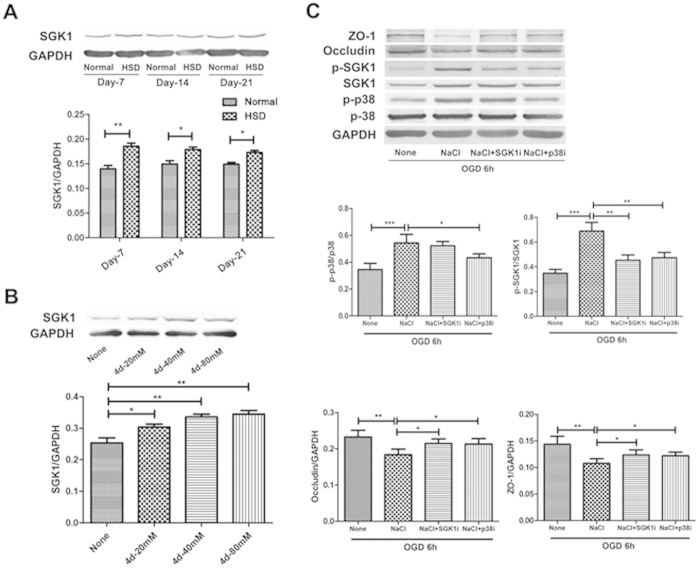
ZO-1 and Occludin reduction induced by NaCl was depended on p38/MAPK and SGK1 signaling. (**A**) SGK1 expression in the ischemic brain hemisphere after HSD. The bar graph shows a significant increase in HSD-treated mice compared with mice on a normal diet. (n = 5 per group; Normal vs HSD, *P < 0.05, **P < 0.01). (**B**) Detection of SGK1 expression in bEnd.3 cells NaCl treatments at different concentrations. The data are pooled from three independent experiments. (*P < 0.05, **P < 0.01). (**C**) Western blotting analysis of ZO-1 and Occludin in bEnd.3 cell after 6 h OGD in the presence or absence of NaCl, p38i or SGK-1i. The bar graphs below show the statistical analysis. (*P < 0.05, **P < 0.01, ***P < 0.001). Values are mean ± SD.

Next, we studied the potential molecular mechanism that was involved in NaCl mediated down-regulation of TJ protein expression. It has been shown that NaCl can enhance hypertonicity through p38/MAPK in mammals^21^. We hypothesized that the high Na^+^ concentration led to the phosphorylation of p38/MAPK that was capable of activating other downstream targets, including SGK1. We first examined the expressions of ZO-1, Occludin, p-SGK1, SGK1, p-p38/MAPK and p38/MAPK under high-salt condition both in *in vivo* and *in vitro* assays. We found that SGK1 expression was significantly increased under high-salt condition both *in vivo* (Fig. 6A, Day-7: *P* = 0.0020, Day-14: *P* = 0.0128 and Day-21: *P* = 0.0161) and *in vitro* (Fig. 6B, None vs 4 d-20 mM: *P* = 0.0341, None vs 4 d-40 mM: *P* = 0.0041 and None vs 4 d-80 mM: *P* = 0.0035). Next, we investigated whether p38/MAPK/SGK-1 pathway was responsible for the down-regulation of TJ proteins mediated by NaCl. Pharmacological inhibition of either p38/MAPK (NaCl vs NaCl + p38i: *P* = 0.0194) or SGK1 (NaCl vs NaCl + SGK1i: *P* = 0.0012) reversed the effect of NaCl on expression of TJ-associated proteins ZO-1 (NaCl vs NaCl + SGK1i: *P* = 0.0481 and NaCl vs NaCl + p38i: *P* = 0.0388) and Occludin (NaCl vs NaCl + SGK1i: *P* = 0.0209 and NaCl vs NaCl + p38i: *P* = 0.0325) following OGD insult (Fig. 6C).

### High urinary sodium levels were associated with large ischemic lesion in human

To further confirm the relationship between excess dietary salt intake and brain ischemia, we compared the differences of the urinary sodium levels and brain ischemic lesions between stroke patients and healthy controls. Although the univariate analysis revealed that the urine sodium levels were higher in the patients with stroke compared with healthy controls (*P* = 0.0084, odds ratio = 1.007), multivariate analysis with covariate adjustment did not demonstrate any statistically significant difference (Table 1). To further evaluate the independent association between sodium intake and ischemic injury, patients with acute ischemic stroke were divided into two groups according to their lesion sizes (Table 2). We found that urinary sodium levels were associated with the size of brain ischemic lesion (*P* = 0.0341, odds ratio = 1.009).

## Discussion

The present study demonstrated that dietary high sodium intake augmented blood brain barrier dysfunction following cerebral ischemic injuries and that this aggravation of BBB damage was possibly a result of down-regulatory effect of Na cation on endothelial tight junction associated proteins ZO-1 and occludin. Pharmacological inhibition experiments revealed that p38/MAPK-SGK1 signaling contributed to the down-regulation of TJ-associated proteins in cerebrovascular endothelia by sodium cation.

Previous studies have suggested that HSD accelerates spontaneous strokes with BBB disruption in rats^22^, and HSD induced hypertension is thought to play a pivotal pathogenic role^23^. However, our high salt diet mice model based on previous research^3^ demonstrated no alterations in systolic blood pressure compared to normal diet controls. Together with *in vitro* studies of divalent magnesium ion and mannitol, our results suggested that exacerbation of BBB damage by sodium cation was most likely independent of blood pressure and osmotic factors. Clinical data demonstrated a positive correlation between urinary sodium and ischemic lesion size in stroke patients. Despite that urinary sodium does not necessarily accurately reflect dietary intake, it is not irreasonable to speculate that sodium intake might have a similar correlation pattern.

The acute inflammatory response after focal cerebral ischemia is characterized by the influx of peripheral leukocytes into the cerebral parenchyma^24^,^25^. The access of leukocytes into the brain is normally blocked by the BBB^26^,^27^,^28^. Under pathological conditions, leukocytes can adhere to activated post-capillary venules and infiltrate the brain parenchyma across the BBB, which was consistent with our findings that the infiltrations of peripheral macrophages (CD11b^+^CD45^high^Ly6G^−^), neutrophils (CD11b^+^CD45^high^Ly6G^+^) and CD4^+^ T cells were detected in the ischemic brain hemispheres, and HSD-treated mice appeared a higher level of infiltration of leukocytes as shown in Fig. 3.

The serum Na^+^ concentration *in vivo,* which is approximately 140 mM, is similar to the Na^+^ concentration in the standard cell culture media. However, it is less well appreciated that considerably higher Na^+^ concentrations between 160 mM and even as high as 250 mM can be encountered in the interstitium^3^,^29^. Given the principle that extracellular fluids and intracellular compartments are in constant equilibrium, the excess interstitial Na^+^ is considered to be readily mobilized into the bloodstream^28^, and vascular endothelial cells throughout the body, including the BMECs, could encounter this high Na^+^ environment. Because the brain microvascular endothelial cells are more vulnerable and sensitive to any homeostatic alterations, the higher sodium environment will, in theory, affect the properties of the BMECs.

Brain microvascular endothelial cells (BMECs) differ from the other vascular beds due to their high expression of TJ proteins^30^. Complex intercellular tight junctions restrict molecular and cellular exchange between the CNS and associated blood vessels, rendering the high selectivity of the blood brain barrier^31^. The primary TJ proteins expressed in BMECs are Occludin, claudins, ZO-1, and cingulin, which help to tether the TJ proteins to the actin cytoskeleton, and the reduction of TJ protein levels disrupts TJ integrity and increases the BBB permeability^32^. As observed in Fig. 3 that high sodium conditions enhanced peripheral leukocytes infiltration, and this was accompanied by a marked decrease in the expression TJ proteins (Occludin and ZO-1) in BMECs (Fig. 2B,C). No changes were observed between sham-operated mice on a normal and high-salt diet groups, indicating that all the differences promoted by the HSD only occurred under cerebral ischemic conditions.

Interestingly, BBB leakage did not continue to increase as the duration of HSD prolonged as assessed by EBD staining and the expression of ZO-1 and Occludin displayed a similar pattern as shown in Fig. 2A and Fig. 2C. The underlying mechanisms still need to be further investigated. Nonetheless, in the absence of any evidence of hypertension, changes in TJ proteins and infiltrating inflammatory cells (Fig. 1D, *p* > 0.05) suggest that acceleration of BBB integrity damage might be one of the mechanisms contributing to the HSD-induced aggravation of cerebral injuries. Moreover, this failure of the BBB, attributed to the loss of TJ protein function and expression in the BMECs, probably occurred directly via the elevated serum Na^+^ concentration (Fig. 1C), which was sufficient to cause endothelial function changes under ischemic conditions.

Based on these findings, we then examined the molecular pathway underlying the reduction in TJ proteins induced by high-salt addition. Excessive SGK1 expression and activity participate in the pathophysiology of several disorders, including obesity, thrombosis^33^, stroke^14^, inflammation^34^ and tumor growth^35^. Moreover, excessive salt intake up-regulates SGK1 expression, promoting the development of SGK1-related diseases^3^. Ye Zhang *et al.* have shown that SGK1 is primarily expressed by microglial cells and endothelial cells in the brain^11^, which is consistent with our finding that the total ischemic brain tissue in HSD mice had higher SGK1 expression compared with that in normal mice (Fig. 6A). In addition, *in vitro* assays with different concentrations of NaCl stimulation revealed that bEnd.3 cells displayed increased SGK1 expression in a dose-dependent manner (Fig. 6B), confirming that the high-salt manipulation enhanced SGK1 expression both *in vivo* and *in vitro*.

Hypertonic stress in mammals is sensed through the p38/MAPK pathway, and the yeast hypertonic stress-response element and SGK1 activation are regulated by p38/MAPK^21^. Our results confirmed previous finding that (Figure S3) that high-salt conditions lead to a significant increase in p38/MAPK phosphorylation, which activates other downstream targets, including SGK1^3^. Therefore, we proposed that increased NaCl concentration leads to the down-regulation of TJ proteins through the phosphorylation of p38/MAPK and the subsequent activation of SGK1. To confirm the involvement of this pathway, SB202190 (p38 inhibitor) and GSK650394 (inhibitor of SGK1) were selected in this study. Under OGD conditions, ZO-1 and Occludin expression in bEnd.3 cells were decreased by 80 mM NaCl incubation, whereas 4 μM SGK1 inhibitor or p38 inhibitor weakened this effect. Hence, we concluded that the p38/MAPK-SGK1 signaling pathway was involved in the mechanism of the TJ protein alterations under high-salt and OGD conditions.

In summary, our data suggested a hypertension-independent role of dietary salt intake in the progression of ischemic stroke and provides evidence for clinical practice in the management of patients with cerebral ischemic diseases.

## Methods

### Mice, high-salt diet and pMCAL induction

Male C57/BL6 mice (weighing 20–25 g) were obtained from the Peking Vital River Laboratory Animal Ltd (Beijing, People’s Republic of China) and maintained in accordance with the guidelines of the Care and Use of Laboratory Animals published by the China National Institute of Health. Furthermore, all experiments were approved by the Harbin Medical University Ethics Committee. A total of 300 mice were used in our experiment, and included randomly divided into Sham + normal diet group (Sham), Sham + high-salt siet group (Sham + HSD), high-salt diet for 7 days + pMCAL group (Day-7 HSD), high-salt diet for 14 days + pMCAL group (Day-14 HSD), high-salt diet for 21 days + pMCAL group (Day-21 HSD), high-salt diet for 28 days + pMCAL group (Day-28 HSD). Mice received normal chow and tap water (normal group) or sodium-rich chow containing 4% NaCl and tap water containing 1% NaCl (high-salt diet, HSD) for 7 days, 14 days, 21 days and 28 days. Then, pMCAL was induced as previously described^36^. Briefly, mice were anesthetized with 2% Nembutal, and then a vertical incision was made between the left eye and ear. A horizontal incision on the temporal muscle was made using spring scissors, and the middle cerebral artery (MCA) was exposed by removing the small pieces of skull with bone rongeurs. Finally, the distal portion of the MCA was ligated using a small vessel cauterizer. Ultimately, the pMCAL mouse model was established, and in the sham-operated group, the arteries were blunt dissected without ligation.

### Noninvasive blood pressure

We used a computerized tail-cuff system (BP2010A, softron, China) to perform the non-invasive systolic blood pressure measurement. Measurements were conducted for 4 inconsecutive days (on day-7/14/21/28) prior to pMCAL induction during the feeding of the selected diets^37^.

### Urine and serum sodium concentrations

After 7 days, 14 days, 21 days and 28 days of the selected diet, 24-hour urine and serum samples were collected prior to pMCAL induction using a metabolic cage system (Sebiona, China), and the sodium concentrations were measured by a Modular DPP Automatic Biochemical Analyzer (Roche Diagnostics, Shanghai, China).

### TTC staining

The mice were euthanized 2 days after the pMCAL procedure. The brains were rapidly removed after an intracardial infusion of PBS and then cut along the coronal suture into 2 mm slices using a brain slicer. Brain slices were immediately incubated in 2,3,5-triphenyltetrazolium chloride (TTC) solution (2% solution in PBS) for 30 minutes at 37 °C. Areas not stained red with TTC were considered to be damaged.

### Evans blue extravasation

In brief, a 4% solution of EBD (4 ml/kg of body weight) was injected intraperitoneally and allowed to circulate for 2 hours two days after pMCAL. Under deep anesthesia, mice were perfused with saline until colorless fluid outflowed from the right atrium. Then, ischemic cerebral hemispheres were collected after decapitated. The brain specimens were weighed (wet weight of each sample was 50 mg), homogenized in 1 ml 50% trichloroacetic acid, and centrifuged at 15,000 × *g* for 20 minutes. Then, 0.5 ml of the resultant supernatant was added to 1.5 ml anhydrous ethanol for a colorimetric assay using a fluorescence spectrophotometer (Ex620 nm, Em680 nm) to obtain the OD value to determine the EBD concentration. The EBD content (per mg of wet weight) within the brain tissue was used to determine the BBB permeability rate of EBD.

### Flow cytometry analysis

Briefly, leukocytes from the ischemic brain hemisphere in different groups were isolated by a 70%–30% Percoll gradient centrifugation 2 days post pMCAL induction and analyzed by flow cytometry with the staining of CD4-FITC, CD45-PerCP, CD11b-APC, and Ly6G-PE antibodies (all Abs and isotype matched controls were purchased from BD Biosciences, San Diego, CA, USA).

### Cell culture

The immortalized murine brain microvascular endothelial cell line bEnd.3 (ATTC, Manassas, VA, USA), which retains key features of cerebral endothelial function, was used for the *in vitro* assay^38^,^39^. The cells were cultured in Dulbecco’s modified Eagle’s medium (DMEM) supplemented with 10% fetal bovine serum (FBS), 100 IU/mL penicillin, and 100 IU/mL streptomycin. After reaching 90% confluence, the medium was replaced by DMEM with 5% FBS containing NaCl at concentrations of 0/20/40/80/160 mM. Additionally, 2 and 4 μM of SGK1 inhibitor (SB202190, Tocris Bioscience, United Kingdom) and p38 inhibitor (1, 2 and 4 μM, GSK650394, Tocris Bioscience, United Kingdom) were added in the culture medium together with 80 mM NaCl for analyzing the p38/MAPK/ SGK1 signaling pathway.

### Western blotting

bEnd.3 cells and ischemic mice brains were homogenized with RIPA lysis buffer (Santa Cruz Biotechnology, Santa Cruz, CA, USA). Supernatants were collected after centrifuging at 12,000 × *g* for 15 min at 4 °C, followed by protein concentration measurement using the BCA protein assay kit (Pierce, Rockford, IL, USA). Samples were separated on 4–10% Tris-HCl Ready SDS-polyacrylamide gels (Bio-Rad Laboratories, Hercules, CA, USA) and incubated with various antibodies: anti-ZO-1 (Zymed Laboratories, 1:500), anti-GAPDH and anti-β-actin antibodies (Santa Cruz, 1:2,000), anti-occludin (Abcam, 1:1,000), anti-SGK-1 (Abcam, 1:1,000) and p-SGK1 (Abcam, 1:500), anti-p38 and anti-p-p38 (Cell Signaling Technology, 1:1,000). All secondary antibodies were purchased from Santa Cruz. The data were normalized to levels of β-actin or GAPDH, and the degree of immunoreactivity was expressed relative to the corresponding control.

### Immunofluorescence staining

Brain cryosections 14 μm thick were prepared for immunofluorescence staining. An incubation of CD31/ZO-1/Occludin antibodies (Abcam, 1:200), followed by a FITC-conjugated goat-anti-rabbit IgG or TRITC-conjugated goat-anti-rat IgG (Invitrogen, 1:500) was conducted. Finally, sections were incubated in 4′,6-Diamidino-2-phenylindole dihydrochloride (DAPI, Sigma-Aldrich) for 5 min, mounted and observed under a confocal microscope (Zeiss, Germany).

### Permeability assays

Permeability assays were conducted as previously described^40^. The bEnd.3 cells (1 × 10^4^) were plated on top of 3-μm pore-size upper Transwell (Corning, USA) chambers with different doses of NaCl (0/20/80 mM) for 4 days, and then medium was substituted with glucose-free DMEM containing 50 μg/ml of FITC-BSA (Sigma) to the Transwell upper chambers at the time of 6 h OGD treatment. Then, media (both 100 μl) were obtained from the upper and lower chambers of each well, and the fluorescence intensity was measured using a FL600 microplate fluorescent reader (Biotek). The diffusion rate of the bEnd.3 cells was calculated as follows: [BSA lower chamber] × 100/[BSA upper chamber]. Non-OGD groups were cultured in a cell incubator (5% CO_2_) in glucose-free DMEM.

### Oxygen Glucose Deprivation

The OGD condition was determined as described previously. Briefly, bEnd.3 cells were initially exposed to OGD medium (glucose-free DMEM), placed in an anaerobic chamber (Pla sLabs, MI, USA) containing 95% nitrogen and 5% carbon dioxide at 37 °C for a 6 h incubation. The supernatants and cell extracts were collected after OGD for the following experiments.

### Human urine sample collection and measurements

Human research was approved by the Harbin Medical University Ethics Committee, and all participants signed informed consent forms. The study was performed in accordance with the guidelines of clinical medical research in Harbin Medical University. From October 2013 to April 2014, 97 patients were enrolled within 6 hours after acute ischemic stroke at the Department of Emergency, First Affiliated Hospital of Harbin Medical University. Excluding cerebrovascular diseases, 110 healthy controls were selected from the medical Examination Center of Harbin Medical University from August 2012 to August 2013. Detailed demographic characteristics of these patients are listed in Table S1. The participants involved in this study were not suffering from liver diseases, kidney diseases or any other metabolic diseases. After fasting for 12 h, urine samples were collected, centrifuged at 2,000 × g for 10 min to remove impurities, and then frozen immediately and stored at −80 °C until analysis. The sodium and potassium concentrations in each urine specimen were determined using a Modular DPP Automatic Biochemical Analyzer (Roche Diagnostics, Shanghai, China).

### Statistical analysis

Numerical data are expressed as the mean ± SD. One-way or two-way ANOVA was performed for group analyses followed by Tukey’s *post hoc* comparison. Univariate and multivariate analysis with a covariate-adjusted logistic regression model were used to evaluate the association between sodium levels and stroke. All statistical tests were two-tailed, and *p*-values < 0.05 were considered statistically significant. Statistical analyses were performed using SAS version 9.3 (SAS Institute, Inc).

## Additional Information

**How to cite this article**: Zhang, T. *et al.* Excess salt exacerbates blood-brain barrier disruption via a p38/MAPK/SGK1-dependent pathway in permanent cerebral ischemia. *Sci. Rep.* **5**, 16548; doi: 10.1038/srep16548 (2015).

## Supplementary Material

Supplementary Information

## Figures and Tables

**Table 1:** Multivariate analysis with covariate adjustment between the patients with ischemic stroke and healthy controls.

	Odds ratio	95% CI^a^	*p*-value^b^
Sodium excretion	1.007	1–1.014	0.0522
Age	1.159	1.104–1.217	<0.0001
Gender	0.901	0.407–1.993	0.7968
Hypertension	4.844	2.213–10.606	<0.0001
Diabetes	1.306	0.439–3.887	0.6308
Cardiovascular disease	0.45	0.137–1.48	0.1887

^a^CI, confidence interval.

^b^*p*-values were calculated based on a multivariate logistic regression model with covariates adjustment.

**Table 2:** Association between lesion size and demographic characteristics of stroke patients.

Characteristics	Small lesion^a^	Large lesion^b^	Total	p-value^c^
Age (years)				0.4005
≤60	21	31	52	
**>**60	22	23	45	
Gender				0.6063
Male	30	35	65	
Female	13	19	32	
Hypertension				0.4503
Absent	20	21	41	
Present	23	33	56	
Diabetes				0.2518
Absent	36	40	76	
Present	7	14	21	
Cardiovascular disease				0.4127
Absent	39	46	85	
Present	4	8	12	

^a^Lesion size ≤1 cm.

^b^lesion size >1 cm.

^c^*p*-values were derived using a two-tailed Chi-squared test.
