# Supplementary material for: Excess salt exacerbates blood-brain barrier disruption via a p38/MAPK/SGK1-dependent pathway in permanent cerebral ischemia
Source: Sci Rep. 2015 Nov 9;5:16548. doi: 10.1038/srep16548 (PMC4637879; doi:10.1038/srep16548)
Supplement: Supplementary Information [file srep16548-s1.doc]

**Excess salt exacerbates blood-brain barrier disruption via a p38/MAPK/SGK1-dependent pathway in permanent cerebral ischemia**

Tongshuai Zhang^1^; Shaohong Fang^2^; Cong Wan^1^; Qingfei Kong^1^; Guangyou Wang^1^; Shuangshuang Wang^1^; Haoqiang Zhang^1^; Haifeng Zou^1^; Bo Sun^1^; Wei Sun^3^; Yao Zhang^1^; Lili Mu^1^; Jinghua Wang^1^; Jing Wang^3^; Haiyu Zhang^4^; Dandan Wang^1*^; Hulun Li^1*^

^1^ Department of Neurobiology, Harbin Medical University, Harbin, China 150081

^2^ The Key Laboratory of Myocardial Ischemia, The Second Affiliated Hospital of

Harbin Medical University, Harbin, China 150081

^3^ Department of Neurology, The First Affiliated Hospital of Harbin Medical

University, Harbin, China 150081

^4^Department of Epidemiology and Biostatistics, Harbin Medical University, Harbin,

China 150081

***Corresponding Author:**

Hulun Li

Dept of Neurobiology, Harbin Medical University, Harbin, China 150081

Lab phone: +86 451 8666 2943

Fax: +86 451 8750 2363

Email: lihulun@aliyun.com

Dandan Wang

Dept of Neurobiology, Harbin Medical University, Harbin, China 150081

Email: wangddhmu@gmail.com


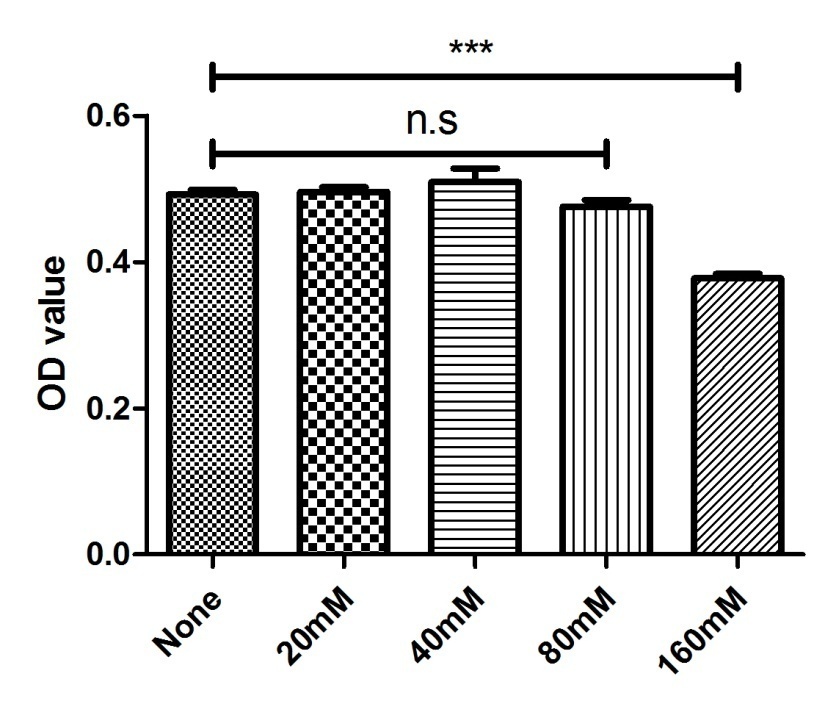


**Figure S1 Cell viability measurements performed using MTT-assay.** bEnd.3 cells were cultured with different concentration of NaCl for 4 days. Data are presented as mean ± SD. *** P < 0.001..


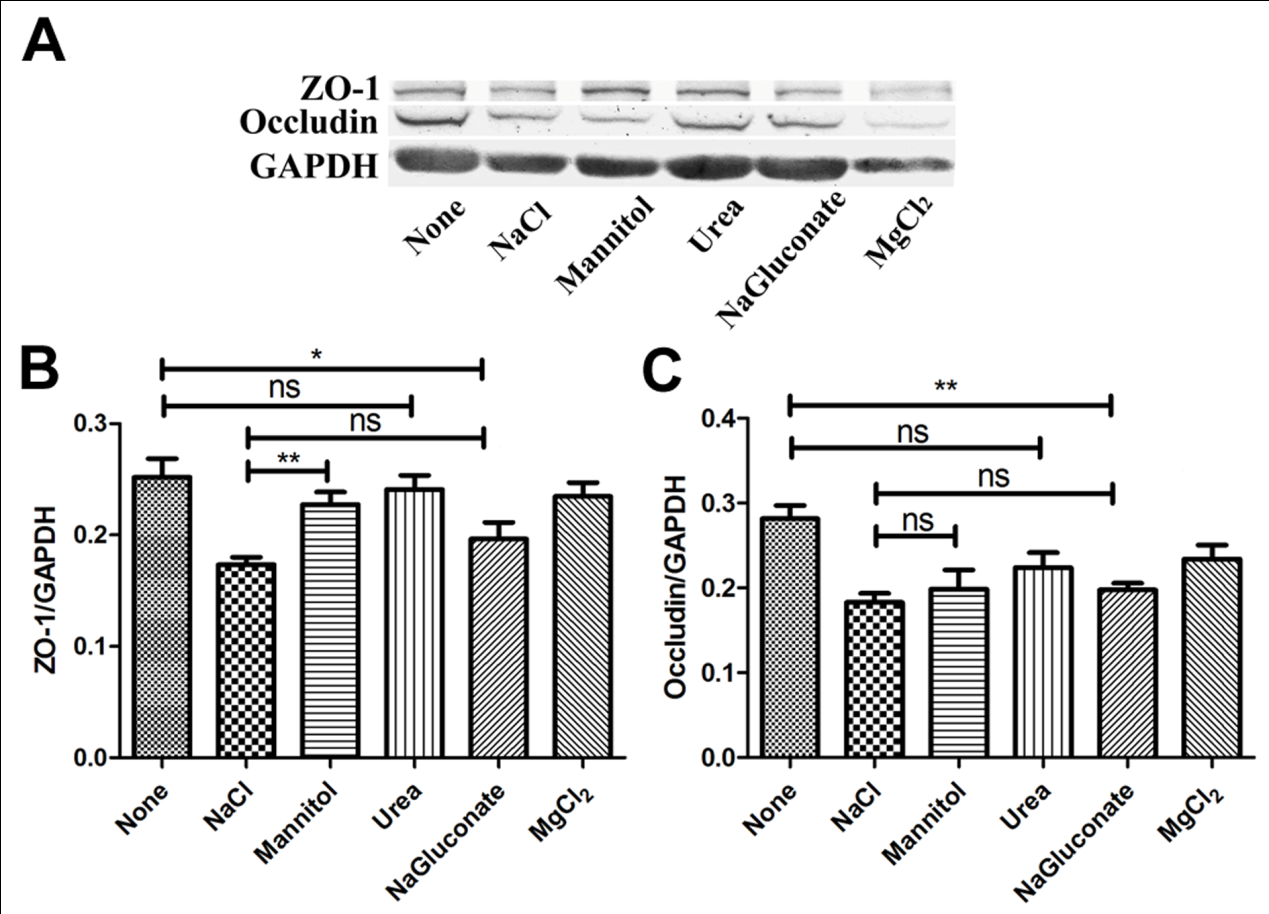


**Figure S2 Effects of various stimuli on the expression of ZO-1 and Occludin in bEnd.3 cells**. **(A)** The bEnd.3 cells were treated with 40 mM NaCl, 80 mM mannitol, 80 mM urea, 40 mM sodium gluconate and 26.7 mM MgCl_2_, and the expression levels of ZO-1 and Occludin were observed by western blot. **(B, C)** Bar graphs showing the quantification of ZO-1 and Occludin expression. Data are presented as mean ± SD. * P < 0.05, ** P < 0.01..


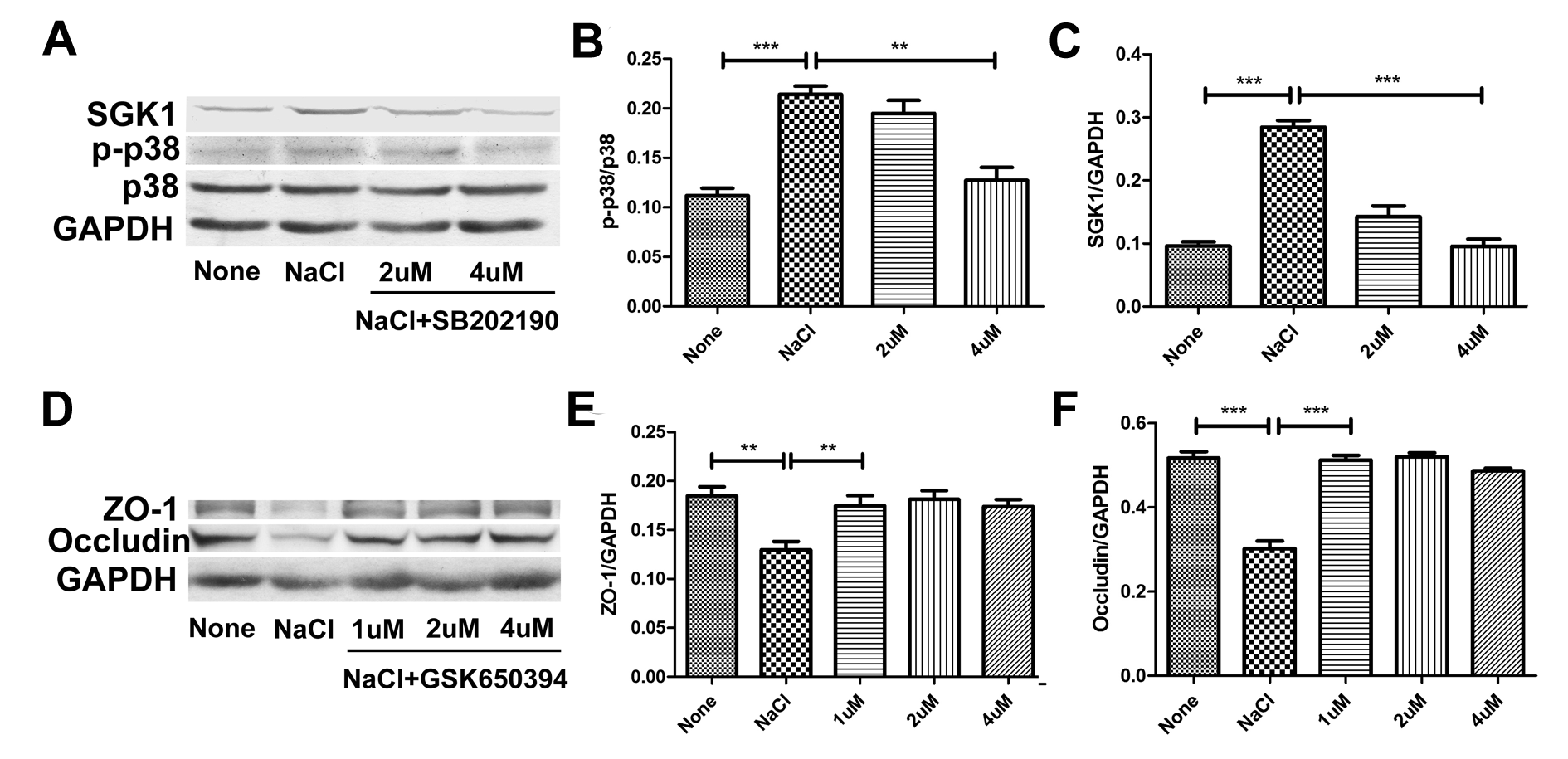


**Figure S3 ZO-1 and Occludin reduction induced by NaCl depends on p38/MAPK and SGK1 in normal condition. (A)** Cell extracts were collected from the bEnd.3 cells cultured with NaCl (0 mM, 80 mM), NaCl (80 mM) plus 2 or 4 μM p38i. Western blotting was processed for the detection of p38, p-p38 and SGK1 expression. **(B, C)** Bar graph showed the quantification analysis of SGK1 expresssion and phosphorylation of p38. **(D)** Cells extracts from bEnd.3 cells cultured with NaCl (0 mM, 80 mM), NaCl (80 mM) plus 1, 2 or 4 μM SGK1i. ZO-1 and Occludin expression were detected by western blotting. **(E, F)** Bar graph showed quantification analysis of ZO-1 and Occludin expression. SGK1 inhibitor inhibited the NaCl induced ZO-1 and Occludin reduction. Data are presented as mean ± SD. ** P < 0.01, *** P < 0.001..

**Table S1 Detailed demographic characteristics of patients with stroke and healthy controls**

| Characteristics | Control | Stroke |
| --- | --- | --- |
| Sodium excretion (mmol/L) (median, range) | 102.0, 25.0-241.0 | 121.0, 24.0-265.0 |
| Potassium excretion (mmol/L) (median, range) | 36.8, 8.5-104.4 | 20.4, 6.6-72.0 |
| Age (years) (median, range) | 50, 30-72 | 59, 39-91 |
| Gender |  |  |
| Male (no.) (%) | 74 (67.27) | 65 (67.01) |
| Female (no.) (%) | 36 (32.73) | 32 (32.99) |
| Body-mass index (median, range) | 24.5, 17.8-31.0 | 22.9, 17.8-28.8 |
| Educational level |  |  |
| Less than high-school (no.) (%) | 10 (9.09) | 54 (55.67) |
| College (no.) (%) | 92 (83.64) | 41 (42.27) |
| Postgraduate or more (no.) (%) | 6 (7.27) | 2 (2.06) |
| Tobacco use |  |  |
| Absent (no.) (%) | 21 (19.09) | 42 (43.3) |
| Present (no.) (%) | 89 (80.91) | 55 (56.7) |
| Alcohol consumption |  |  |
| Absent (no.) (%) | 27 (24.55) | 27 (27.84) |
| Present (no.) (%) | 83 (75.45) | 70 (72.16) |
| Blood pressure (mm Hg) |  |  |
| Systolic (median, range) | 130.5, 98.0-160.0 | 146.0, 103.0-242.0 |
| Diastolic (median, range) | 83.0, 59.0-108.0 | 86.5, 60.0-128.0 |
| Hypertension |  |  |
| Absent (no.) (%) | 89 (80.91) | 41 (42.27) |
| Present (no.) (%) | 21 (19.09) | 56 (57.73) |
| Diabetes |  |  |
| Absent (no.) (%) | 102 (92.73) | 76 (78.35) |
| Present (no.) (%) | 8 (7.27) | 21 (21.65) |
| Cardiovascular disease |  |  |
| Absent (no.) (%) | 102 (92.73) | 85 (87.63) |
| Present (no.) (%) | 8 (7.27) | 12 (12.37) |

**Table S2: The mean and SD per group for animal experiments were listed as follows:**

**Table S2 A**. The mean value and SD for Figure 1

| Day | Infarct Volume (mm^3^) | |  | Serum Na^+^ (mM/L) | |  | U_Na_V (μmol/24h) | |
| --- | --- | --- | --- | --- | --- | --- | --- | --- |
|  | Normal | HSD |  | Normal | HSD |  | Normal | HSD |
| 7 | 20.9±1.55 | 23.55±0.98 |  | 154.16±4.64 | 158.84±2.19 |  | 103.26±13.36 | 576.46±89.64 |
| 14 | 20.13±1.14 | 22.63±0.91 |  | 154.29±4.70 | 159.14±1.49 |  | 98.28±10.87 | 602.29±113.93 |
| 21 | 21.40±1.15 | 23.48±1.00 |  | 154.13±4.51 | 158.90±1.66 |  | 102.13±11.85 | 579.10±107.66 |
| 28 | 21.13±1.21 | 23.65±1.10 |  | 153.61±4.31 | 158.68±1.64 |  | 95.53±7.66 | 591.48±98.22 |

**Table S2 B**. The mean value and SD for Figure 2

| Day | EB Leakage (ng/mg) | |  | Occludin/GAPDH | |  | ZO-1/GAPDH | |
| --- | --- | --- | --- | --- | --- | --- | --- | --- |
|  | Normal | HSD |  | Normal | HSD |  | Normal | HSD |
| sham | 15.48±4.10 | 16.80±4.32 |  | 0.57±0.05 | 0.58±0.04 |  | 0.33±0.02 | 0.34±0.02 |
| 7 | 79.23±14.56 | 160.64±19.403 |  | 0.38±0.03 | 0.30±0.02 |  | 0.20±0.02 | 0.09±0.02 |
| 14 | 72.53±17.62 | 132.14±35.98 |  | 0.38±0.02 | 0.32±0.02 |  | 0.20±0.02 | 0.16±0.02 |
| 21 | 78.79±14.05 | 109.90±19.88 |  | 0.38±0.02 | 0.34±0.01 |  | 0.19±0.03 | 0.18±0.02 |
| 28 | 72.67±25.78 | 126.72±22.44 |  | - | - |  | - | - |

**Table S2 C**. The mean value and SD for Figure 3

| Day | %CD11b+CD45highLy6G^-^ | |  | %CD11b+CD45highLy6G^+^ | |  | % of CD4+ T cell | |
| --- | --- | --- | --- | --- | --- | --- | --- | --- |
|  | Normal | HSD |  | Normal | HSD |  | Normal | HSD |
| sham | 2.91±0.31 | 3.42±0.55 |  | 1.45±0.48 | 1.55±0.34 |  | 0.20±0.05 | 0.21±0.08 |
| 7 | 18.48±2.96 | 37.08±2.51 |  | 6.15±1.30 | 12.08±0.73 |  | 3.97±1.18 | 12.01±2.00 |
| 14 | 18.90±1.82 | 25.73±4.96 |  | 5.96±0.85 | 9.90±1.43 |  | 5.27±0.92 | 7.76±1.13 |
| 21 | 17.30±1.35 | 19.75±1.36 |  | 6.24±1.25 | 8.15±0.81 |  | 4.77±0.71 | 6.49±0.93 |

**Table S2 D**. The mean value and SD for Figure 4 A and Figure 6 B

| Group | Occludin/GAPDH | ZO-1/GAPDH | SGK1/GAPDH |
| --- | --- | --- | --- |
| None | 0.26±0.02 | 0.12±0.01 | 0.25±0.03 |
| 4d-20mM | 0.25±0.02 | 0.13±0.01 | 0.30±0.02 |
| 4d-40mM | 0.22±0.01 | 0.11±0.01 | 0.34±0.02 |
| 4d-80mM | 0.20±0.02 | 0.09±0.01 | 0.34±0.02 |

**Table S2 E**. The mean value and SD for Figure 4 B

| Group | Occludin/GAPDH | ZO-1/GAPDH |
| --- | --- | --- |
| None | 0.32±0.02 | 0.21±0.01 |
| 80mM-Day-2 | 0.28±0.02 | 0.19±0.01 |
| 80mM-Day-4 | 0.24±0.01 | 0.16±0.01 |
| 80mM-Day-6 | 0.23±0.02 | 0.15±0.01 |

**Table S2 F**. The mean value and SD for Figure 5B-D

| Group | Occludin/GAPDH | ZO-1/GAPDH |
| --- | --- | --- |
| None | 0.40±0.02 | 0.16±0.01 |
| NaCl | 0.36±0.01 | 0.14±0.01 |
| None+OGD | 0.29±0.02 | 0.13±0.01 |
| NaCl+OGD | 0.23±0.01 | 0.11±0.01 |

**Table S2 G**. The mean value and SD for Figure 5A

| Group | Non-OGD | OGD |
| --- | --- | --- |
| 0mM | 3.41±0.56 | 20.29±2.23 |
| 20mM | 3.56±0.42 | 21.21±2.14 |
| 80mM | 4.96±0.57 | 30.16±2.53 |

**Table S2 H**. The mean value and SD for Figure 6A

| Group | None | HSD |
| --- | --- | --- |
| Day-7 | 0.14±0.01 | 0.19±0.01 |
| Day-14 | 0.15±0.01 | 0.18±0.01 |
| Day-21 | 0.15±0.01 | 0.17±0.01 |

**Table S2 I**. The mean value and SD for Figure 6C

| Group | None | NaCl | NaCl+SGK1i | NaCl+p38i |
| --- | --- | --- | --- | --- |
| p-p38/p38 | 0.34±0.05 | 0.54±0.06 | 0.52±0.03 | 0.43±0.03 |
| p-SGK1/SGK1 | 0.35±0.03 | 0.69±0.07 | 0.45±0.04 | 0.47±0.04 |
| Occludin/GAPDH | 0.23±0.02 | 0.18±0.02 | 0.21±0.01 | 0.21±0.01 |
| ZO-1/GAPDH | 0.14±0.01 | 0.11±0.01 | 0.12±0.01 | 0.12±0.01 |
